# Evaluation of Multi-Objective Optimization Algorithms for NMR Chemical Shift Assignment

**DOI:** 10.3390/molecules26123699

**Published:** 2021-06-17

**Authors:** Emel Maden Yılmaz, Peter Güntert, Şima Etaner-Uyar

**Affiliations:** 1 Department of Computer Engineering, Faculty of Computer and Informatics Engineering, İstanbul Technical University, Maslak, 34469 İstanbul, Turkey; yilmazeme15@itu.edu.tr; 2 ETH Zürich, Laboratory of Physical Chemistry, ETH Zürich, Vladimir-Prelog-Weg 2, 8093 Zürich, Switzerland; 3 Center for Biomolecular Magnetic Resonance, Institute of Biophysical Chemistry, Goethe University Frankfurt am Main, Max-von-Laue-Str. 9, 60438 Frankfurt am Main, Germany; 4 Department of Chemistry, Graduate School of Science and Engineering, Tokyo Metropolitan University, Hachioji, Tokyo 192-0397, Japan; 5 Department of Computer Engineering, İstanbul Ayvansaray University, Prof. Muammer Aksoy Cad. No: 10 Kazlıçeşme, Zeytinburnu, 34020 İstanbul, Turkey; simauyar@ayvansaray.edu.tr

**Keywords:** NMR, chemical shift assignment, automated assignment, multi-objective optimization

## Abstract

An automated NMR chemical shift assignment algorithm was developed using multi-objective optimization techniques. The problem is modeled as a combinatorial optimization problem and its objective parameters are defined separately in different score functions. Some of the heuristic approaches of evolutionary optimization are employed in this problem model. Both, a conventional genetic algorithm and multi-objective methods, i.e., the non-dominated sorting genetic algorithms II and III (NSGA2 and NSGA3), are applied to the problem. The multi-objective approaches consider each objective parameter separately, whereas the genetic algorithm followed a conventional way, where all objectives are combined in one score function. Several improvement steps and repetitions on these algorithms are performed and their combinations are also created as a hyper-heuristic approach to the problem. Additionally, a hill-climbing algorithm is also applied after the evolutionary algorithm steps. The algorithms are tested on several different datasets with a set of 11 commonly used spectra. The test results showed that our algorithm could assign both sidechain and backbone atoms fully automatically without any manual interactions. Our approaches could provide around a 65% success rate and could assign some of the atoms that could not be assigned by other methods.

## 1. Introduction

The field of three-dimensional structure analysis is a crucial interdisciplinary research area in natural sciences to detect the structure, function, and interactions of biological macromolecules [1]. NMR is one of the methods to determine protein and nucleic acid structure [2]. The sequence-specific assignment of the resonance frequencies or chemical shifts of ^1^H, ^13^C, and ^15^N nuclei is a central step of the NMR method and plays an essential role in the successful interpretation of NMR experiments [3]. The computer programs that calculate the protein structure from NMR data require almost completely assigned chemical shifts of the atoms to produce an accurate protein structure [4,5]. Even if there are many software programs to automate the chemical shift assignment process [6,7], a considerable amount of time by an experienced spectroscopist is needed if the determination is performed manually or by a semi-automated method.

There have been many different studies on the chemical shift assignment problem and these approaches demonstrate different aspects by using different methodologies of computer science, including a probabilistic interaction network of evidence algorithm [8], methods derived from artificial intelligence [9], and a peak-particle dynamics simulation algorithm [10]. Some of these methods used simulated annealing in the research [11]. Exhaustive search algorithms have been employed for small and medium-sized proteins [12,13]. Some of the existing approaches adopted heuristic algorithms [14,15,16]. There exist also some hybrid solutions in which evolutionary algorithms [17] are combined with local optimization methods [18,19]. Some of these approaches focused only on a partial solution of the problem, the backbone assignment, and exempted a chemical shift assignment of the atoms on the sidechain of the molecules [9,11,20,21]. However, both sidechain and backbone chemical shift assignments are required for determining a complete three-dimensional structure of a protein [2]. Even if the assignment of both sidechain and backbone atoms is carried out, some of the existing methods [12,13,14,22] require long computation times and do not apply well to proteins of large size. They can only produce results for small or medium-sized macromolecules.

In this paper, we present a novel algorithm that provides a fully automatic assignment of the chemical shift values of NMR experiments to its respective atoms. We conducted a deep dive into the problem from the computer science point of view and analyzed the objectives along with the nature of the problem to find a suitable problem model. We investigated several different types of evolutionary algorithms, including multi-objective optimization methods. Our method assigns chemical shift values to the hydrogen, carbon, and nitrogen nuclei without any manual interactions and any additional input data required from the user. Additionally, our solution produces a complete assignment for the whole protein in the experiment, where all of the sidechain and backbone atoms are taken into consideration for a complete resonance frequency assignment.

We implemented our algorithms in a standalone program and compared its results against two state-of-the-art automated resonance assignment programs, FLYA [19] and PINE [8]. The program CYANA [23] is a fully automatic solution to calculate the three-dimensional structure of the protein [24]. CYANA includes all of the steps of NMR experiments such as data processing, peak picking, chemical shift assignment, NOE assignment, and structure calculation. The chemical shift assignment step of CYANA is realized with the FLYA algorithm that has been applied for a variety of different NMR assignment problems [7,19,25,26,27,28,29,30,31]. The program PINE assigns chemical shift values automatically by using evidence estimates from empirical distributions and consistency measures. We applied our algorithms, FLYA and PINE, to several different experimental datasets. Here, we present the results of these comparisons along with the conclusions and further work. 

## 2. Methods

The NMR chemical shift assignment comprises assigning the chemical shift values measured in spectra to individual atoms of the molecule. This chemical shift assignment step is a cumbersome process and requires computational intelligence to deal with the challenges of the problem. Chemical shift assignment provides a basis for the three-dimensional structure calculation programs. Even supposing that the structure calculation program performs very well, it is impossible to produce correct results using wrong chemical shift assignments as input. Chemical shift assignments are, thus, crucial information for the complete NMR structure determination or other NMR studies of proteins. Here, we consider the chemical shift assignment problem assuming as given the other steps of the protein determination such as peak picking, NOE assignment and structure calculation. 

### 2.1. Input and Output Files

The first input file of our algorithm is the *sequence file* that contains the amino acid sequence and the residue numbering of the protein under investigation. The second input file is the CYANA *library file* that contains the atoms, their connectivity, the covalent geometry for each residue, as well as the (formal) magnetization transfer pathways for each spectrum type that define the pattern of atoms that are expected to give rise to a peak in the spectrum with a given probability [19]. Atom combinations that match with these predefined patterns generate the *expected peaks* for each spectrum. The CYANA program provides commands to calculate these expected peaks, which are transferred directly from CYANA into our algorithm. As a third type of input files, our algorithm needs the *measured peak* file for each spectrum that includes the type of the spectrum and the positions of the peaks identified in this specific spectrum, given in the format of the program XEASY [32].

The output of our algorithm is a list of chemical shift values assigned to the atoms of the protein. 

### 2.2. Assignment Algorithms

The chemical shift assignment problem searches for solutions in a large search space, where an exhaustive search for the optimal solution(s) is very hard and in general not feasible. Therefore, instead of systematic search methods, we applied heuristic approaches to this problem. We also separated the objectives of the problem from each other and transformed the NMR assignment problem into a multi-objective optimization problem structure. Since evolutionary algorithms already work with a set or population of solution candidates, they appeared to be a suitable match for the multi-objective optimization problems due to their population-related approach.

We implemented our solution using the MOEA framework [33], which is an open-source Java library of multi-objective optimization algorithms. We designed this problem model on the MOEA library with an object-oriented programming approach in Java language.

We used the non-dominated sorting genetic algorithm II (NSGA2), the non-dominated sorting genetic algorithm III (NSGA3), and the genetic algorithm (GA) of the MOEA framework for this work. In addition, we developed and tested a hill-climbing algorithm to improve the results.

The NSGA2 algorithm [34] is summarized in Algorithm 1 [35]. The NSGA2 algorithm is a multi-objective evolutionary optimization algorithm that uses elitism and crowded comparison operators to keep diversity. All non-dominated individuals are classified into a layer and these individuals are then separated from the population. Afterwards, another non-dominated group is created from the remaining individuals of the population. This process continues until all individuals belong to a certain non-dominated layer. A dummy fitness value is assigned to each layer, which is called a non-dominated rank. This value increases in lower layers, ensuring that the individuals from the first layer are more likely to reproduce. Furthermore, niching is applied by calculating a crowding distance for each individual. The crowding distance represents the density of solutions surrounding a particular solution. Its insertion into the algorithm ensures diversity. Selection is based on both random rank and crowding distance by a so-called crowded comparison operator. In case two solution candidates are from different fronts, the one with the lower rank is selected. If they are in the same front, then the one from the less crowded region is preferred, which ensures that solution candidates from less crowded regions are proposed preferentially to be selected by the algorithm.
**Algorithm 1** NSGA-II algorithm [35].$1:\;\mathrm{Procedure}\;\mathrm{NSGA}-\mathrm{II}\;(N^{\prime },g,f_{k\;}\left(X_{k}\right))\;\nabla N\prime \;\mathrm{members}\;\mathrm{evolved}\;g\;\mathrm{generations}\;\mathrm{to}\;\mathrm{solve}\;f_{k\;}X$2: Initialize population ℙ′3: Generate random population—size N′4: Evaluateobjectivevalues5: Assign rank (level) basedonParetodominance-sort6: Generate child population7:  Binarytournament selection8:  Recombinationandmutation9: Fori=1togdo10:  Foreachparentandchildinpopulationdo11:   Assignrank(level)basedonPareto-sort$12:\;\;\;\mathrm{Generat}\mathrm{sets}\mathrm{of}\mathrm{nondominated}\mathrm{vectors}\mathrm{along}{\mathrm{PF}}_{\mathrm{known}}$13:   Loop(inside)byaddingsolutionstonextgenerationstartingfromthe  firstfrontN′individualsfound determinecrowdingdistancebetween  pointsonfront14:  Endfor15:  Selectpoints  (elitist)onlowerfront (lower rank)andare  outsideacrowdingdistance16:  Createnextgeneration17:   Binarytournamentselection18:   Recombinationand mutation19: Endfor20: Endprocedure

The NSGA3 [36] algorithm is an improved version of the NSGA2 algorithm. It promises good results especially on the many-objective optimization problems, which are the optimization problems with four or more objectives. The pseudo-code for the creation process of one generation in the algorithm is presented in Algorithm 2 [36]. No explicit selection operator exists in this method. Reference directions are created in objective space and individuals that best represent these references are selected.

The GA [37] algorithm in the MOEA framework is a standard genetic algorithm that uses a single objective optimization approach with elitism. Even if all of the four objectives are defined in the description of the problem, there is only one objective in the GA. All given objectives are combined into a single score function with the same weight to obtain one single objective value.
**Algorithm 2** Generation *t* of NSGA-III procedure [36].Input:HstructuredreferencepointsZsorsupplied aspirationpointsZa,parent  populationPt$\mathrm{Output}:P_{t+1\;}$$1:\;\;S_{t\;}=\emptyset ,\;\;i=1$$2:\;\;Q_{t\;}=\;\mathrm{Recombination}+\mathrm{mutation}\;(P_{t\;})$$3:\;\;R_{t}=P_{t\;}\cup Q_{t\;}$$4:\;(F_{1\;},F_{2\;},\ldots )=$ $\;\mathrm{Non}-\mathrm{dominated}-\mathrm{sort}\;(R_{t\;})$5: Repeat$6:\;S_{t\;}=S_{t}\cup F_{i}\;and\;i=i+1$$7:\;\mathrm{Until}\;\left|S_{t}\right|\geq N$$8:\;\mathrm{Last}\;\mathrm{front}\;\mathrm{to}\;\mathrm{be}\;\mathrm{included}:\;F_{l\;}=F_{i}$$9:\;\mathrm{If}\;\left|S_{t}\right|=N\;\;\mathrm{then}$$10:\;P_{t+1\;}=S_{t}\mathrm{break}$11:Else$12:\;P_{t+1}\;=\cup_{j=1}^{i-1}F_{j}$$13:\;\mathrm{Points}\;\mathrm{to}\;\mathrm{be}\;\mathrm{chosen}\;\mathrm{from}\;F_{l\;}:K=N-\left|P_{t+1}\right|$$14:\;\mathrm{Normalize}\;\mathrm{objectives}\;\mathrm{and}\;\mathrm{create}\;\mathrm{reference}\;\mathrm{set}\;Z^{r}:Normalize\;\left(f^{n},\;S_{t},Z^{r},Z^{s},Z^{a}\right)$$15:\;\mathrm{Associate}\;\mathrm{each}\;\mathrm{member}\;s\;\mathrm{of}\;S_{t}\mathrm{with}a\mathrm{reference}\mathrm{point}:$$\;\;\;\;\left[\pi \left(s\right),\;d\left(s\right)\right]=Associate\left(S_{t},Z^{r}\right)\;\;\%\;\pi \left(s\right):closest\;reference\;point,$$\;\;\;\;\;\;\;d:distance\;between\;s\;and\;\pi \left(s\right)$$16:\;\mathrm{Computer}\;\mathrm{niche}\;\mathrm{count}\;\mathrm{of}\;\mathrm{reference}\;\mathrm{point}\;j\;\epsilon \;Z^{r}:\rho_{j}=$$\;\;\underset{s\varepsilon S_{t}/F_{l}}{\sum }\left(\left(\pi \left(s\right)=j\right)?1:0\right)$$17:\;\mathrm{Choose}\;K\;\mathrm{members}\;\mathrm{one}\;\mathrm{at}\;a\;\mathrm{time}\;\mathrm{from}\;F_{l}\mathrm{to}\mathrm{construct}$$\;\;P_{t+1}:Niching\;\left(K,\;\rho_{j},\pi ,d,Z^{r},F_{l},P_{t+1}\right)$18Endif

### 2.3. Objectives and Constraints

The score function of these algorithms plays an important role in the success of the results. In order to define the scoring function of the problem, four fundamental characteristics of a correct assignment are defined as the objective values of the problem [10,19]: shift normality, alignment, completeness, and low degeneracy. Shift normality aims to keep the chemical shift values of the atoms within the statistical distributions that are derived from a large number of assigned proteins and stored in chemical shift libraries [38]. The alignment objective keeps the corresponding chemical shift values of all observed peaks assigned to a given atom aligned with each other as much as possible. The goal of the completeness objective is to assign as many peaks as possible. The last objective is the low degeneracy, which means that degenerate peaks are allowed but their number should be small. Degenerate peaks are expected peaks that are assigned to an observed peak to which more than one expected peak is assigned. The mathematical representation of these objectives is given in Section 2.4.

The constraints, as defined in [19], are taken as the constraint in our work. These are the following: (1) An expected peak can be assigned to a single observed peak. It is not possible to map one expected peak to more than one observed peak. (2) Expected peaks can be mapped to observed peaks of the same spectrum only. It is not allowed to assign an expected peak of one spectrum to an observed peak of another spectrum. These two constraints are already covered by the problem representation. (3) The variance of the chemical shifts assigned to an atom cannot exceed the given tolerance representing the accuracy of the measurement. This constraint is already covered by the alignment objective above, so it is removed from the problem model. As a result, all of these constraints are already covered by the problem representation, so there are no explicit constraints implemented in the problem model.

### 2.4. Score Functions

The score functions for the multi-objective algorithms are derived from the global score function *G* of FLYA [19], given in Equation (1).

(1) $$G=\frac{\sum_{a\in A}\left[w_{1}\left(a\right)Q_{1}\left(a\right)+\sum_{n\in N{’}_{a}}w_{2}\left(a,n\right)Q_{2}\left(a,n\right)/b\left(n\right)\right]}{\sum_{a\in A_{0}}\left[w_{1}\left(a\right)+\sum_{n\in N_{a}}w_{2}\left(a,n\right)\right]}$$

This function evaluates the aforementioned four objectives of an entire protein’s assignment and attributes a global score value to that assignment. The numerator sums up the score contributions from each assigned atom. The denominator is used to normalize the *G* values. *A*_0_ in the denominator refers to the set of all atoms involved in expected peaks. *N_a_* refers to the set of expected peaks of atom *a*. The numerator of this equation calculates the score of the assignment by evaluating the objective value results of the assigned atoms. *A* in the numerator denotes the set of assigned atoms, so *A* is a subset of *A*_0_. The contribution of each assigned atom, *a,* is calculated and summed up in the numerator to form the global score, *G,* of the assignment of the protein. *N’_a_* denotes the set of expected peaks of the atom *a* that are mapped to a measured peak. The value *b*(*n*) represents the ambiguity of the assignment and it is equal to the number of expected peaks that are mapped to the same measured peak as the expected peak *n. Q*_1_(*a*) evaluates the shift normality of the assigned atoms, meaning the alignment of the assigned chemical shift values of the atoms to their general chemical shift statistics. Similarly, *Q*_2_(*a*,*n*) evaluates the alignment of the chemical shift position of atom *a* in peak *n*, to the other chemical shift values for the same atom defined in other mapped expected peaks of the spectrum. As there are many objectives of the chemical shift assignment problem, an aggregating function is used in this equation to produce a single global score value. In the calculations of FLYA, the weight values *w*_1_(*a*) = 4 and *w*_2_(*a*,*n*) = 1 are used to combine the objective value scores, as defined in the original FLYA publication [16].

The four objectives of the problem are covered by this score function. For the purpose of multi-objective optimization, we separated the global score *G* into four different score functions *F*_1_, …, *F*_4_ in our multi-objective algorithms, where each of these score functions represents one objective of the problem that is to be minimized:

(2) $$F_{1}=-\underset{a\in A}{\sum }Q_{1}\left(a\right),\;\;\;F_{2}=-\underset{a\in A}{\sum }\underset{n\in {N^{\prime }}_{a}}{\sum }Q_{2}\left(a,n\right),\;\;F_{3}=-\left|A\right|,\;\;F_{4}=\underset{a\in A}{\sum }\underset{n\in {N^{\prime }}_{a}}{\sum }b\left(n\right)$$

The first objective is the negative sum over all shift normality values, which is represented by *Q*_1_(*a*). The second objective is the negative sum over the alignment of the chemical shift references from different peaks, which is shown by *Q*_2_(*a*,*n*). These objectives are calculated by the same logic as in FLYA [19]. The third objective is the completeness objective that is the number of assigned atoms, which is implicitly covered by the FLYA global score function. In our model, we added an explicit third objective to evaluate the total number of atoms assigned in an assignment of the protein, |*A*|. The fourth objective is the sum over the degeneracy of the atoms. The multi-objective algorithms try to minimize these four objectives. Note the minus signs in *F*_1_, *F*_2_, *F*_3_, since shift normality, alignment, and completeness should be maximized, whereas degeneracy, represented by *F*_4_, should be minimized. We did not use weight values such as *w*_1_(*a*) and *w*_2_(*a*,*n*) in our representation.

### 2.5. Problem Representation

**Figure 1:**
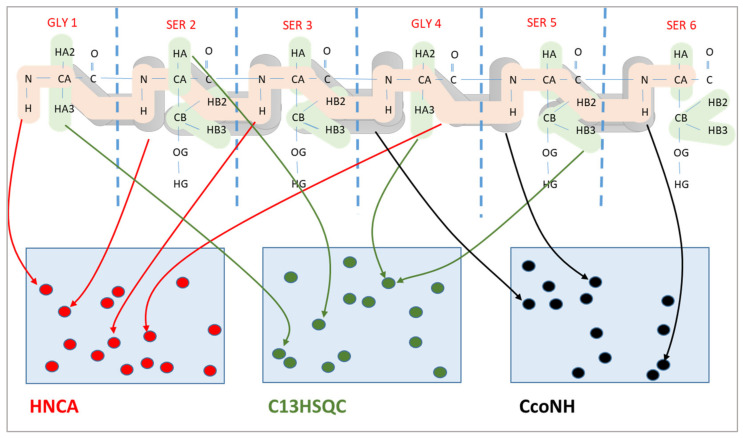
Chemical shift assignment by mapping expected peaks (colored atom patterns in the upper part) to the measured peaks (in the lower part) in three different spectra.

The chemical shift assignment problem is a combinatorial optimization problem, where the expected peaks are mapped to the measured peaks in multiple spectra. An example of three spectra is shown in Figure 1. The upper part of the figure shows the expected peaks, where it is always known precisely which atoms are involved in a peak. However, their location is available only very approximately. Similarly, we have the measured peaks of each spectrum, which are shown in the lower part of the figure. Here, the specific locations of these peaks in the spectrum are known; however, the atom pairs giving rise to these peaks are unknown. The chemical shift assignment maps expected peaks to measured peaks. Since the atoms belonging to each expected peak and the chemical shift coordinates of each measured peak are known, this mapping allows to assign chemical shift values to atoms of the protein.

**Figure 2:**
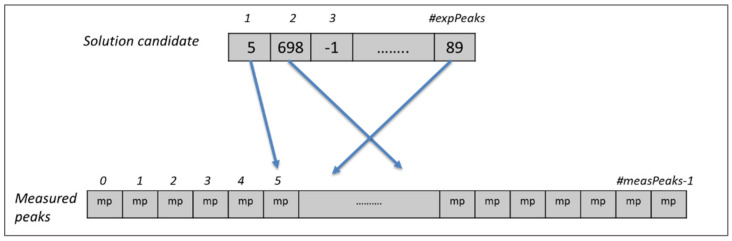
Solution candidate and measured peaks for one spectrum. The solution candidate represents a chromosome of our problem model, where each gene shows the expected peak that is assigned to that single measured peak. A value of −1 (e.g., for expected peak 3) indicates that the expected peak is not assigned to any measured peak.

As shown in Figure 1, expected peaks are always assigned to measured peaks of the same spectrum. Evolutionary algorithms work not with a single assignment solution for the protein but with a population that is composed of multiple solution candidates (“chromosomes”) made up by individual peak mappings (“genes”). In our representation, each solution candidate is given by a list of the expected peaks with their mapping to a measured peak, or the absence of such a mapping. Each spectrum is handled separately in our representation. Figure 2 shows a chromosome for one of the spectra. 

**Figure 3:**
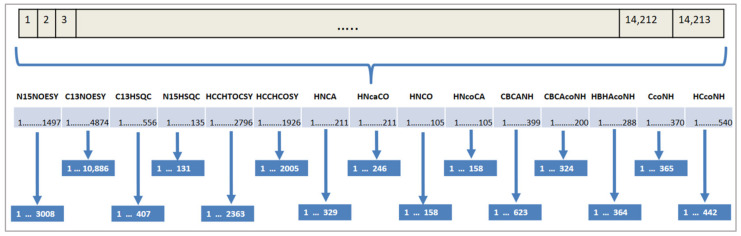
Chemical shift assignment representation for fifteen spectra.

Each gene of the chromosome represents an expected peak in that spectrum and the values in the cells show the index of the corresponding measured peak if the expected peak is assigned to any, or −1 to indicate the absence of a mapping. If there is more than one spectrum, then the solution candidate is expanded for the expected peaks of the other spectra as shown in Figure 3. In this example, there are fourteen spectra and each expected peak of these spectra is appended one after another to create the solution candidate, which forms the chromosome for the evolutionary algorithm. Each expected peak of a spectrum can be assigned only to measured peaks of the same spectrum. For this reason, each expected peak from each spectrum is represented by one gene in the chromosome of a solution candidate. This approach covers automatically the first constraint of the problem since it is not possible to assign more than one value to an expected peak in the chromosome. Similarly, the second constraint is also covered naturally, since mappings are conducted only between expected and measured peaks in the same spectrum.

**Figure 4:**
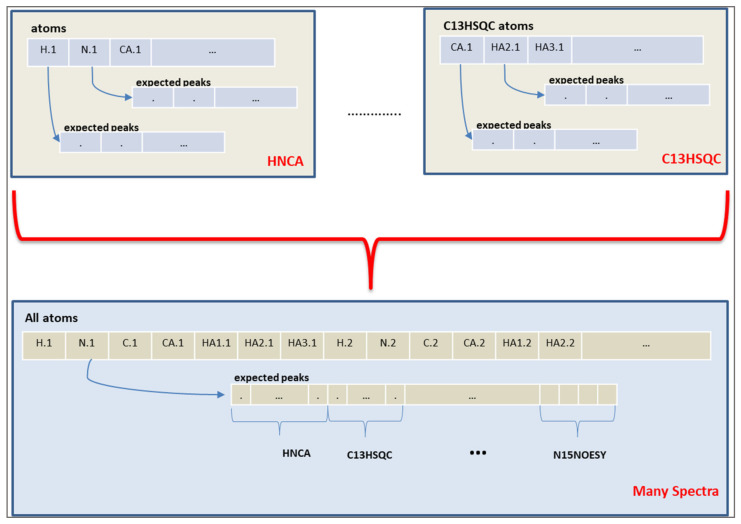
Atoms of an expected peak. The upper part shows the atom representations for two spectra, HNCA and C13HSQC in this example. The lower part shows the atom representation for the expected peaks in multiple spectra.

The chromosome of the algorithm represents the expected peaks of the experiments. A peak assignment maps one expected peak to a measured peak of that spectrum. However, the objective values are calculated from the chemical shift values of each atom, instead of the peak mappings. Therefore, a connection from the peak mapping to chemical shift values of atoms was established by using the fact that each expected peak involves several atoms depending on the spectrum definition. An atom representation is built and all expected peaks from different spectra are assigned to the atoms, as shown in Figure 4.

### 2.6. Further Improvements in the Algorithms

We produced the initial population of solutions for all algorithms with the following constructive logic in order to start with a better population than a randomly created one. Logic very similar to the local optimization and search space reduction of FLYA [19] was applied here. We reduced the search space during the initial population creation. After the initialization, any offspring in the population is allowed during the evolutionary algorithm iterations and the search space reduction logic is not applied anymore. The initialization logic of the population is implemented by a constructive method. Initially, the search space in each dimension of expected peaks is created with the statistical frequency range for the atom. Additionally, atom type-specific tolerance values of 0.03 ppm for ^1^H, 0.4 ppm for ^13^C, and 0.4 ppm for ^15^N are added to the upper boundaries and subtracted from the lower boundary values of the chemical shift search range of the atoms. These tolerances correspond to the experimental accuracy of peak positions in the input peak lists and are chosen such as to include the chemical shift uncertainties of all assigned atoms. One expected peak among the non-assigned ones is selected randomly. The measured peaks are checked one by one and the first measured peak located in the restricted search area is selected for the assignment. The chemical shift frequencies of the measured peak are added to the measured peak frequency lists of the adjacent atoms. The search spaces of these atoms are updated with the average values of their measured peak frequency lists and the tolerance values. This procedure assures that the next expected peak to be mapped leads to a list entry that is consistent with the previous ones. These steps are explained in Algorithm 3. The procedure continues until all expected peaks have been evaluated and relevant assignments performed. This approach ensures that the completeness objective is fulfilled as much as possible. Then, the individual is added to the initial population and the next individual is created following the same logic evaluating the expected peaks in a different random order until all individuals of the population are created. In our experiments, we had fifty individuals in each population.**Algorithm 3** Constructive initialization approach.Input:Expected peaks and measured peaksOutput:Initial population1: Select randomly one expected peak to be assigned2: Check possible measured peak candidates 3: Get the first fitting measured peak from the search space4: Update search space with the new assignment5: If there is still expected peak to be evaluated for assignment6:  Go back to step #1to evaluateanewexpectedpeak7: Endif8: Add the individual to the initial population9: If  the population is not complete   then10:  Go back to step #1 to create a new individual11: End if 

To keep diversity in the population of solutions, optionally some of these individuals can be created randomly in different proportions between 0 and 100% of the population.

### 2.7. Crossover and Mutation Rates

The crossover rate and mutation rate represent the probability of applying a crossover operator and a mutation operator to the individuals in an evolutionary optimization method, respectively. We applied different crossover rates and mutation rates in our experiments. Either a dynamic crossover rate, 1/*L*, can be used, where *L* represents the length of the individual, i.e., the number of expected peaks. Alternatively, static crossover rates of 0.001, 0.01, 0.1, and 1.0 can be used. In the same way, different mutation rates can be applied in the algorithms. 

### 2.8. Repetition Parameter

We ran our algorithms several times and consolidated the output [19] to obtain the final results. A new parameter, the repetition parameter, specifies how many individual executions of one algorithm are performed using different random numbers. The overall result of one run of the algorithm is composed of the collection of the results of all repetitions, which is obtained by chemical shift consolidation [19]. With an increase in the repetition parameter, the results may be improved. However, the runtime of the calculation increases as well, approximately linearly with the repetition parameter. 

### 2.9. Hill-Climbing and Hyper-Heuristic Algorithms

Hill-climbing algorithms search for local optima in their neighborhood. They try to find an improved neighbor at each step. We implemented a hill-climbing algorithm [39] from scratch in the MOEA framework that checks each expected peak of the assignment and searches for a better measured peak in its neighborhood. We used the global score function of Equation (1), which is the aggregation of all objectives. The hill-climbing algorithm was combined with the NSGA2, NSGA3, and GA algorithms to find better local optima. To this end, a hyper-heuristic algorithm was implemented in the MOEA framework that combines the selected multi-objective algorithm with the hill-climbing algorithm. Optionally, each iteration of the NSGA2, NSGA3 or GA algorithms can be followed by one iteration of the hill-climbing algorithm.

### 2.10. Problem Challenges

Defining and finding the optimal correspondence between expected and observed peaks in a set of NMR spectra for a protein is not trivial. The main reason for this is that measured NMR spectra are not perfect: in general, they do not contain all peaks that one would expect to see. In addition, they also contain noise and artifact peaks, which do not correspond to an expected peak. Furthermore, for some NMR experiments, it is difficult to predict theoretically the expected peaks. Therefore, the notion of “optimal correspondence” should be expressed in a scoring function that is defined in a robust way such that it tolerates a significant amount of missing observed peaks (i.e., expected peaks for which a corresponding observed peak could not be found) and artifacts (i.e., observed peaks that do not correspond to any expected peak). It is well possible in practice that only about 50% of the expected peaks can be observed as, for instance, in the Csp A [7] and ENTH [40] proteins, and that the list of observed peaks contains as many artifact peaks as real ones as, for instance, for the NS-1 [7] and ENTH [40] proteins. If defined properly, the maximal score value corresponds to the best, i.e., most correct, resonance assignment.

**Figure 5:**
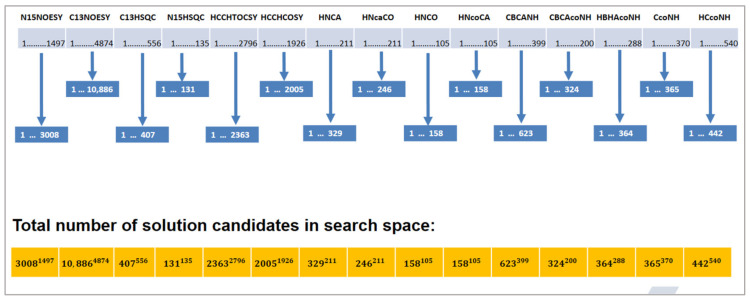
Assignment on several spectra. The upper part shows the number of the expected peaks and measured peaks for each spectrum. The lower part below shows the total number of solution candidates for the whole problem.

Additionally, the search space for our problem is huge. Each expected peak of a spectrum can be assigned to each measured peak of that spectrum. Figure 5 shows an example of the total number of solution candidates. The first spectrum in this example is N15NOESY with 1497 expected peaks and 3008 measured peaks. This spectrum creates 3008^1497^ solution candidates. The number of solution candidates from other spectra is calculated as shown in Figure 5. The whole search space is composed of the combinations of these solution candidates, which is a huge number.

## 3. Results

We performed our tests on several different datasets, for which the chemical shift values have previously been assigned by conventional methods. We assumed that these results are correct and compared our results to these reference values. The manually determined chemical shift assignment values were used only to evaluate the quality of the automated assignment.

The algorithms were run five times independently and their results consolidated for the final chemical shift assignments. There were 50 individuals in each population of our algorithms. The initial populations were created by the constructive initialization approach. The crossover and mutation rates were set to 1.0. The repetition parameter was set to 10. In some of the experiments below, we changed these default parameters for testing. The iteration parameter specifies how many times an evolutionary algorithm reproduces its population. The iteration parameter was set to 10,000 in our experiments. Unless mentioned otherwise, these default values were used in the tests. 

We compared our results against the FLYA [19] and PINE [8] programs. These programs are capable of determining automatically backbone and side-chain chemical shift assignments. The FLYA program already exists as the default solution for the chemical shift assignment problem in the CYANA framework [41]. We used the I-Pine webserver from http://i-pine.nmrfam.wisc.edu (accessed on 15 April 2021) for PINE [42]. The same sequence file and peak lists were used for all comparisons between the algorithms. FLYA was run with its default value, 20, of independent runs that are consolidated into a consensus assignment. PINE was run with its default values via the webserver. 

The chemical shift assignments are grouped as correct, wrong, or unassigned with respect to the chemical shift values from the reference lists that were obtained by conventional techniques. The *correct* chemical shift values show the number of atoms whose deviations from their reference chemical shift values are within the experimental tolerance values of 0.03 ppm for ^1^H, 0.4 ppm for ^13^C, and 0.4 ppm for ^15^N. Deviations larger than these tolerances are marked as *wrong*. The *unassigned* atom counts show the number of atoms that were not assigned in those runs with respect to the atoms in the reference lists. The atoms that do not exist in the reference chemical shift list of the experiments were ignored. 

### 3.1. Results on the Fes SH2 Protein

We performed experiments with the NMR dataset of a protein for which an NMR structure determination was completed earlier by conventional methods: the 114-residue Src homology 2 domain from the human feline sarcoma oncogene Fes (Fes SH2) [43,44,45]. We divided this protein into stretches of 30 residues in order to evaluate the behavior of our algorithms in different datasets. As shown in Table 1, we created seven different datasets comprising data from thirty consecutive amino acids each. The starting and ending amino acids 1–10 and 101–114, which include unstructured regions and expression tags, were excluded from the dataset preparation process. We used 11 spectra in each experiment: C13HSQC, CBCANH, CBCAcoNH, CcoNH, HBHAcoNH, HCcoNH, HNCA, HNcaCO, HNCO, HNcoCA, and N15HSQC.

The algorithms were tested on these seven fragments from the Fes SH2 protein. Our algorithms NSGA2, NSGA3, and GA were run five times independently and their mean values are reported in Table 2. 

The numbers in parentheses show the percentages of the correct, wrong and unassigned atoms relative to the total number of chemical shift values in the reference lists that were assigned by conventional techniques. The atoms that were not assigned by conventional techniques were ignored. Over all fragments, our algorithms NSGA2, NSGA3 and GA yielded 62–74%, 7–9% and 63–71% correct assignments, respectively, while the other atoms were assigned incorrectly. FLYA assigned 74–85% of the atoms correctly, 7–11% incorrectly, and 6–19% remained unassigned (without strong assignments [19]). PINE assigned 65–73% of the atoms correctly, 15–20% wrongly, and 11–16% of the atoms remained unassigned. The runtimes of the algorithms were 0.4–0.6 h for NSGA2, 0.5–0.7 h for NSGA3, 0.4–0.6 h for GA, 0.9–1.1 h for FLYA on one core of an Intel Core i7-3720QM 2.60 GHz CPU personal computer, and 0.1 h for PINE on the webserver.

#### 3.1.1. Objective Analysis

The NSGA3 algorithm could not provide as good results as NSGA2 and GA on our datasets. In order to understand the reason for this behavior, we performed a comparison of the objectives as follows. There are four objectives in our problem model: shift normality, alignment, completeness and low degeneracy. Since the MOEA framework can only work with minimization problems, the objectives of Equation (2) are defined with a negative sign, where necessary. The goal of our algorithms is to minimize these objective values.

**Figure 6:**
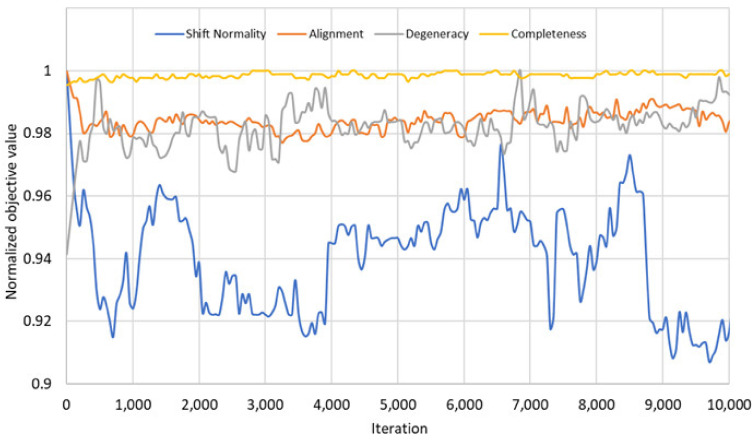
Values of the four different objectives, shift normality (blue), alignment (orange), degeneracy (grey), completeness (yellow), during optimization for the 4th fragment of Fes SH2. Values of each objective are scaled to a maximal value of 1.

For the comparison of the objectives, we checked the values of these new objectives for the 4th fragment of Fes SH2 after each iteration to find possible mutual relationships. The results of these comparisons are given in Figure 6.

These results show that the four objectives of the problem do not always create a trade-off between each other. Figure 6 shows that there are some correlations between the objectives in the initial phase: The objectives shift normality and alignment do not create a trade-off and can be optimized together. Similarly, the objectives degeneracy and completeness can be optimized together since they do not create trade-off either. On the other hand, these two groups of objectives create trade-off against each other. As a result, even if we defined four objectives in our problem model, there are only two competing groups of objectives in the problem that create trade-off. This may be the reason for the NSGA3 results, because the NSGA3 algorithm [25] is designed and optimized for many objective optimization problems, in which there are more than three competing objectives. The correlations between the objectives blurred in the later iterations.

#### 3.1.2. Graphical Representation

**Figure 7:**
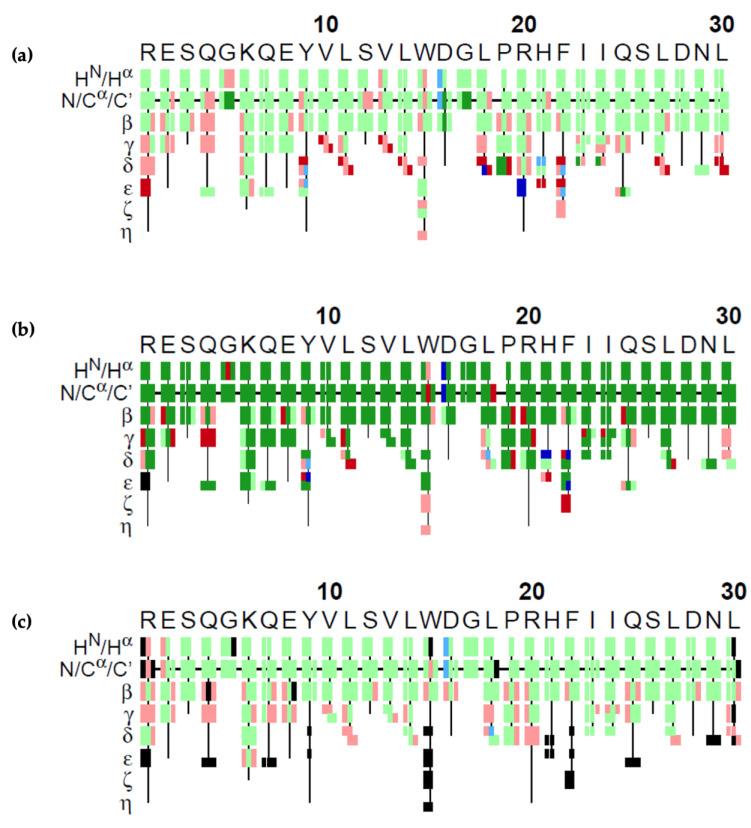
Graphical representation of assignments obtained by the (**a**) GA, (**b**) FLYA, and (**c**) PINE algorithms. Colored rectangles represent the correctness of the assignment of a single atom. Green means that the chemical shift assigned is marked as *correct*, i.e., the difference between the assigned value and the reference value is smaller than the experimental tolerance of 0.03 ppm for ^1^H, 0.4 ppm for ^13^C, and 0.4 ppm for ^15^N. Red means that the assigned chemical shift value is not in that range, i.e., *wrong*. Blue means that the algorithm assigned the atom; however, there does not exist a reference value for that atom to compare. Black indicates that there is a reference chemical shift value for that atom; however, the algorithm did not assign it. In the case of FLYA, dark-colored rectangles indicate that 80% of the 20 independent runs yielded to the same result. The H^N^/H^α^ row shows the atoms H^N^ and H^α^ atoms of the residues. Similarly, the N/C^α^/C’ shows the results for the N, C^α^ and C’ atoms. The lines β, γ, δ, ε, ζ, and η show the assignments of the atoms in sidechain.

The results of our GA algorithm, FLYA and PINE on the 4th fragment are visualized in Figure 7. Each atom is represented by a rectangle and colors represent the correctness of the assignment. Green rectangles show the correct assignments, meaning that the difference between the assigned value and the reference value was less than the experimental tolerance values of 0.03 ppm for ^1^H, 0.4 ppm for ^13^C, and 0.4 ppm for ^15^N. Red colored rectangles show the ones that were assigned wrongly, i.e., with a deviation from the reference value larger than the atom’s tolerance value. GA could make 65% correct assignments. As shown in the figure, the wrong ones mostly lie in the amino acid sidechains of the molecule. FLYA could assign 79% of the atoms correctly. PINE could assign 66% of the atoms correctly on this dataset.

#### 3.1.3. Repetition Parameter Tuning

We checked the impact of the repetition parameter on our experiments. The repetition parameter is set to 10 by default in our calculations. Increasing this number to 50 for the fourth fragment of Fes SH2 yielded the results summarized in Table 3. Our algorithms NSGA2, NSGA3 and GA assigned 70%, 12% and 71% of the atoms correctly, which corresponds to an increase of 1%, 4% and 6% for NSGA2, NSGA3 and GA, respectively. The runtime of all our algorithms increased.

Additionally, we applied a different number of iterations to our algorithms. The iteration parameter is set to 10,000 by default in our experiments. We increased this number to 100,000 and 1,000,000 for the second and the fourth fragments of Fes SH2 and summarized the results in Table 4. The increase in this parameter increased the runtime of each algorithm without significantly improving on the results, indicating that 10,000 iterations suffice for these applications. Due to fluctuations, the best result for a particular case can be found at any of the chosen number of iterations but there is no overall advantage of using a higher number of iterations. Similar fluctuations were also observed in multiple runs with a fixed number of iterations but different random start points.

#### 3.1.4. Mutual Agreement of Assignments from Different Algorithms

We compared the atoms that were assigned correctly by our GA algorithm with the ones that were correctly assigned by FLYA and PINE. We collected the correct assignments of our GA algorithm in this calculation and compared its results against the FLYA and PINE results. We checked the overlapping atoms that were assigned correctly by these algorithms and also the atoms that were assigned only by single algorithms. The results on the fragments are given in Table 5. 

**Figure 8:**
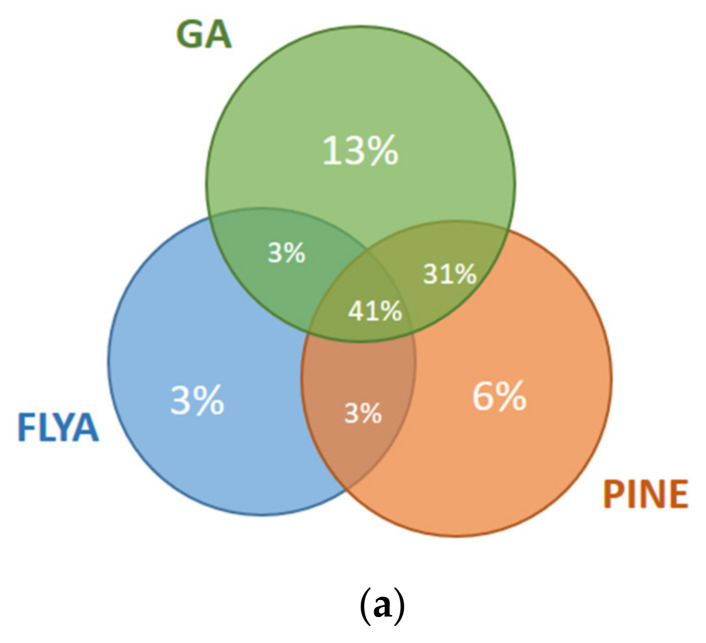

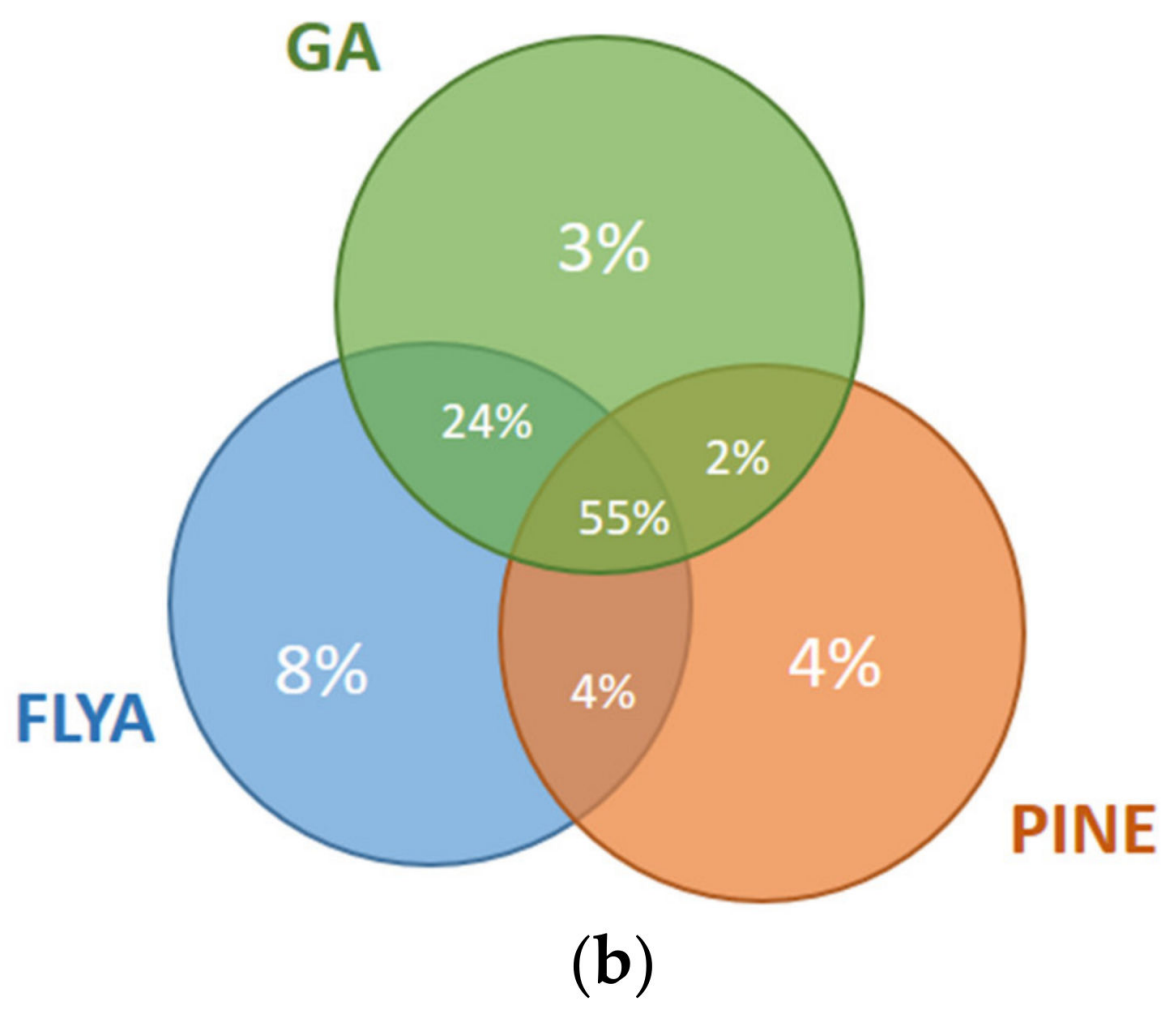
Mutual agreement of correct assignments among different algorithms for the (**a**) 1st fragment and (**b**) 4th fragment of Fes SH2. The percentages of the correct assignments are given relative to the total number of correctly assigned atoms in the corresponding fragment of the Fes SH2 protein.

The results for the first and fourth fragments of the Fes SH2 protein are illustrated in Figure 8. For the first fragment (Figure 8a), 10% of the atoms were assigned correctly only by our GA algorithm but not by PINE or FLYA. A total of 3% of the atoms were assigned correctly only by FLYA and 6% of the atoms only by PINE. Our GA algorithm and FLYA simultaneously assigned 3% of the atoms correctly, where 34% of the atoms were assigned only by PINE and our GA algorithm. A total of 41% of the atoms were assigned simultaneously by our GA algorithm, FLYA and PINE correctly. As seen in the results of the fourth fragment (Figure 8b), 3% of the atoms were assigned correctly only by our GA algorithm but not by PINE or FLYA. A total of 8% of the atoms were assigned correctly only by FLYA and 4% only by PINE. Our GA algorithm and FLYA simultaneously assign 13% of the atoms correctly, where 2% of the atoms were assigned only by PINE and our GA algorithm. A total of 66% of the atoms were assigned correctly simultaneously by our GA algorithm, FLYA and PINE.

### 3.2. Results on the Sso7d Protein

We tested our algorithms on the Sso7d protein from the hyperthermophilic archaebacterium *Sulfolobus solfataricus* that had previously been assigned by conventional methods [46]. There are 65 residues in this protein. We used nine spectra for our experiments: N15NOESY, C13HSQC, C13NOESY, CBCANH, CBCAcoNH, CcoNH, HCcoNH, HNCO and HNcaCO. There are in total 5394 expected peaks and 3527 measured peaks in this dataset.

The algorithms NSGA2, NSGA3 and GA were run five times independently and the mean values of the results are given in Table 6. The numbers in parentheses show the percentages of the correct, wrong, and unassigned atoms relative to the total number of chemical shift values in the reference lists that were assigned by conventional techniques. Atoms that had not been assigned by conventional techniques were ignored.

Our algorithms NSGA2, NSGA3 and GA yielded 67%, 9% and 53% correct assignments, respectively, whereas the other atoms were assigned incorrectly. FLYA assigned 82% and PINE 84% of the atoms correctly. FLYA produced 6% and PINE 9% wrong assignments, while 13% and 7% of the atoms remained unassigned. The runtime was about 7 h for the NSGA2 and NSGA3 algorithms and 5 h for the GA algorithm. FLYA needed 3.3 h and PINE 0.1 h.

## 4. Discussion

The aforementioned results were obtained with the parameter settings of the NSGA2, NSGA3 and GA algorithms that turned out to be optimal in test calculations, which were performed to evaluate the following aspects.

To increase the diversity in the initial population of assignment solutions, we optionally created some of these individuals randomly in different proportions, starting from 0%, by increasing 10% at each step, until 100% of the population is created randomly. However, the best results were achieved with an initial population, in which all individuals are created following the constructive logic explained in Algorithm 3, and none of them is created randomly. This result was expected since the search space of our problem is large, and we have many objectives in our problem. In this case, starting from local optimum individuals is expected to produce better results [47] since the constructively initialized individuals will dominate the random ones in subsequent generations due to their high scores, and the randomly created individuals will diminish after some iterations. Therefore, we continued our tests with initial populations in which all individuals were created with the constructive initialization method.

Likewise, we applied different crossover rates and mutation rates in our experiments, as described in Section 2.7. First, we used dynamic crossover and mutation rates, 1/*L*, where *L* represents the length of the individual, i.e., the number of expected peaks. Alternatively, we applied static crossover and mutation rates of 0.001, 0.01, 0.1, and 1.0. A static crossover rate of 1.0 and a static mutation rate of 1.0 produced the best results and were used for the subsequent calculations.

Values of the repetition parameter that specify how many individual executions of an algorithm are performed using different random numbers were also varied. With an increase in the repetition parameter, the results may, in principle, be improved at the expense of a linear increase in computation time. No significant improvement was obtained with repetition parameter values above 10. For this reason, the repetition parameter was set to 10 in our calculations.

Finally, testing several combinations of hill-climbing and hyper-heuristic algorithms on several different datasets and evaluating their results showed that adding these algorithms does not improve the results. We therefore removed them from the final algorithms.

We tested our algorithms on seven different fragments from the Fes SH2 protein and on the entire Sso7d protein. The results show that our approach can automatically assign a significant part of the sidechain and backbone atoms. The percentage of correct assignments for these fragments by the NSGA2 and GA algorithms was comparable to those from FLYA, whereas the NSGA3 algorithm yielded less than 10% correct assignments. For the seven fragments of the Fes SH2 protein, the FLYA algorithm gave the best results. Additionally, we observed that our algorithms often assigned different atoms than FLYA and PINE, especially in the first fragment of the Fes SH2 protein where 30 atoms were assigned correctly only by our algorithms. On the other hand, the behavior of our algorithms differs when the protein size increases. With an increase in the dataset size, our results remained for the Ssod7 protein approximately 15% below those from FLYA and 17% below the PINE results.

In our NSGA2 and NSGA3 methods, we used the multi-objective approach by separating the objectives of the problem from each other. It turned out that one of the more conventional methods provides better results than the multi-objective approach for these examples. Our approaches work in principle; however, the quality of the results decreases with increasing size of the protein. In further work, the performance of the algorithms might be improved to make them applicable to proteins of larger size, such that these methods will be able to assign complete proteins of all sizes amenable to NMR. Additionally, the three algorithms could be combined to achieve better results and to distinguish atoms that can be assigned consistently and, therefore, more reliably from others with only tentative assignments.

## 5. Conclusions

In this paper, we developed and evaluated evolutionary algorithms based on the MOEA framework of multi-objective optimization algorithms [33] to solve the NMR chemical shift assignment problem. We tested our algorithms on seven different fragments from the Fes SH2 protein and on the entire Sso7d protein. The results show that our approach can automatically assign a significant part of the sidechain and backbone atoms. In principle, multi-objective optimization algorithms can, thus, be applied to the NMR protein assignment problem. However, further research will be needed to obtain results that surpass those of current state-of-the-art single-objective methods.

The main goal of our paper is to evaluate the use of a new class of optimization algorithms, namely multi-objective optimization algorithms, to the protein chemical shift assignment problem in the field of biomacromolecular NMR. To the best of our knowledge, this is the first application of multi-objective optimization algorithms to this problem. Even though at least one existing algorithm based on other principles outperforms our algorithms, multi-objective optimization offers the principal advantage that different objectives do not have to be weighed explicitly against each other as in single-objective methods and that they deliver a set of solutions that are optimal with respect to different combinations of the objectives.

## Figures and Tables

**Table 1:** Experimental peak lists.

Fragment (Residues)	Expected Peaks ^1^	Measured Peaks ^2^	Residues ^3^	Spectra ^4^
1 (11–40)	791	655	30	11
2 (21–50)	817	663	30	11
3 (31–60)	835	648	30	11
4 (41–70)	878	660	30	11
5 (51–80)	816	641	30	11
6 (61–90)	832	661	30	11
7 (71–100)	824	635	30	11

^1^ Number of expected peaks that are used in each fragment. These expected peaks are created by CYANA and given as an input to our algorithms. ^2^ Number of measured peaks used in the experiments. ^3^ Number of residues in each fragment. ^4^ Number of spectra from which peaks were used in each fragment. We used the same spectrum set in each experiment: C13HSQC, CBCANH, CBCAcoNH, CcoNH, HBHAcoNH, HCcoNH, HNCA, HNcaCO, HNCO, HNcoCA, and N15HSQC.

**Table 2:** Chemical shift assignment results for Fes SH2.^1.^

**Fragment**	**Class**	**NSGA2 ^2^**	**NSGA3 ^2^**	**GA ^2^**	**FLYA ^3^**	**PINE ^4^**
1	Correct	238 (67)	25 (7)	245 (69)	274 (77)	245 (69)
	Wrong	118 (33)	331 (93)	111 (31)	38 (11)	54 (15)
	Unassigned	0	0	0	44 (12)	57 (16)
	Run time (h)	0.4	0.5	0.4	1.0	0.1
2	Correct	249 (74)	27 (8)	228 (67)	287 (85)	246 (73)
	Wrong	89 (26)	311 (92)	110 (33)	30 (9)	51 (15)
	Unassigned	0	0	0	21 (6)	41 (12)
	Run time (h)	0.6	0.7	0.6	0.9	0.1
3	Correct	240 (67)	31 (9)	225 (63)	277 (78)	223 (65)
	Wrong	116 (33)	325 (91)	131 (37)	27 (8)	72 (20)
	Unassigned	0	0	0	52 (15)	51 (14)
	Run time (h)	0.5	0.6	0.4	1.0	0.1
4	Correct	252 (69)	31 (8)	237 (65)	287 (79)	240 (66)
	Wrong	113 (31)	334 (92)	128 (35)	26 (7)	69 (19)
	Unassigned	0	0	0	52 (14)	56 (15)
	Run time (h)	0.5	0.6	0.5	1.0	0.1
5	Correct	238 (67)	31 (9)	227 (64)	285 (80)	249 (70)
	Wrong	118 (33)	325 (91)	129 (36)	30 (8)	53 (15)
	Unassigned	0	0	0	41 (12)	54 (15)
	Run time (h)	0.5	0.5	0.4	1.0	0.1
6	Correct	225 (62)	25 (7)	233 (64)	287 (79)	253 (70)
	Wrong	137 (38)	337 (93)	129 (36)	26 (7)	62 (17)
	Unassigned	0	0	0	49 (14)	47 (13)
	Run time (h)	0.5	0.6	0.5	1.0	0.1
7	Correct	240 (67)	31 (9)	252 (71)	265 (74)	246 (69)
	Wrong	117 (33)	326 (91)	105 (29)	24 (7)	71 (20)
	Unassigned	0	0	0	68 (19)	40 (11)
	Run time (h)	0.5	0.6	0.5	1.1	0.1

^1^ Assignment calculations were performed for 7 fragments (Table 1) of Fes SH2 [43,44,45]. The total number of correct, wrong and unassigned atoms are given. Chemical shift assignments are counted as *correct* if the difference between the assigned value and the reference value is smaller than the experimental tolerance values of the atoms, which are 0.03 ppm for ^1^H, 0.4 ppm for ^13^C, and 0.4 ppm for ^15^N. Assignments outside this range are marked as *wrong*. Numbers in the parentheses are the percentages of correct, wrong, and unassigned chemical shift assignments relative to the total number of chemical shift values in the reference lists. ^2^ NSGA2, NSGA3, and GA results are the mean values from 5 independent runs of the algorithm, using 50 individuals in each population. The initial population is created by the constructive initialization approach (see Methods). Crossover and mutation rates are set to 1. The repetition parameter is set to 10. ^3^ FLYA was run with default parameters; 20 independent runs were performed and their results consolidated to a consensus assignment [19]. ^4^ PINE was run with default parameters via its webserver [42]. The run time of the algorithm is the time between input file submission and result arrival.

**Table 3:** Assignment results for the 4th fragment of Fes SH2 for different repetition parameter values.^1.^

	**10 Repetitions**			**50 Repetitions**				
	**NSGA2**	**NSGA3**	**GA**	**NSGA2**	**NSGA3**	**GA**	**FLYA**	**PINE**
Correct	252 (69)	31 (8)	237 (65)	256 (70)	42 (12)	259 (71)	287 (79)	240 (66)
Wrong	113 (31)	334 (92)	128 (35)	109 (30)	323 (88)	106 (29)	26 (7)	39 (19)
Unassigned	0	0	0	0	0	0	52 (14)	56 (15)
Run time (h)	0.5	0.6	0.5	7.2	7.9	6.4	1.0	0.1

^1^ The total number of correct, wrong and unassigned atoms are given. The numbers in parentheses show the corresponding percentages relative to the total number of chemical shift values in the reference list. Except for the repetition parameter, all calculations were performed as detailed in the footnotes of Table 2.

**Table 4:** Chemical shift assignment results for the 2nd and 4th fragments of Fes SH2 for different iteration parameter values.^1.^

Fragment		10,000 Iterations			100,000 Iterations			1,000,000 Iterations				
		NSGA2	NSGA3	GA	NSGA2	NSGA3	GA	NSGA2	NSGA3	GA	FLYA	PINE
2	Correct	249 (74)	27 (8)	228 (67)	238 (70)	24 (7)	216 (64)	244 (72)	26 (8)	221 (65)	287 (85)	246 (73)
	Wrong	89 (26)	311 (92)	110 (33)	100 (30)	314 (93)	122 (36)	94 (28)	312 (92)	117 (35)	30 (9)	51 (12)
	Unassigned	0	0	0	0	0	0	0	0	0	21 (6)	41 (12)
	Run time (h)	0.6	0.7	0.6	1.0	1.3	1.1	6.1	7.5	6.1	0.9	0.1
4	Correct	252 (69)	31 (8)	237 (65)	247 (68)	19 (5)	237 (65)	246 (67)	22 (6)	233 (64)	287 (79)	240 (66)
	Wrong	113 (31)	334 (92)	128 (35)	118 (32)	346 (95)	128 (35)	119 (33)	344 (94)	132 (36)	26 (7)	69 (19)
	Unassigned	0	0	0	0	0	0	0	0	0	52 (14)	56 (15)
	Run time (h)	0.5	0.6	0.5	0.9	1.2	1.2	7.2	7.9	6.4	1.0	0.1

^1^ The total number of correct, wrong, and unassigned atoms are given. The numbers in parentheses show the corresponding percentages relative to the total number of chemical shift values in the reference list. Except for the iteration parameter, all calculations were performed as detailed in the footnotes of Table 2.

**Table 5:** Mutual agreement of correct chemical shift assignments for Fes SH2 among different algorithms.^1.^

Fragment	GA Only	FLYA Only	PINE Only	GA and FLYA	GA and PINE	FLYA and PINE	GA and PINE and FLYA
1	30 (10)	8 (3)	18 (6)	8 (3)	98 (34)	9 (3)	120 (41)
2	5 (2)	24 (8)	5 (2)	28 (9)	6 (2)	30 (10)	205 (68)
3	7 (2)	25 (8)	15 (5)	40 (13)	6 (2)	30 (10)	182 (60)
4	10 (3)	24 (8)	13 (4)	42 (13)	6 (2)	14 (4)	207 (66)
5	7 (2)	25 (8)	13 (4)	30 (10)	6 (2)	32 (10)	198 (64)
6	9 (3)	22 (7)	14 (4)	32 (10)	6 (2)	39 (12)	194 (61)
7	17 (6)	19 (6)	17 (6)	26 (8)	9 (3)	10 (3)	210 (68)

^1^ Assignment calculations were performed for 7 fragments (Table 1) of Fes SH2 [43,44,45]. The total number of correct, wrong and unassigned atoms are given. Chemical shift assignments are counted as correct if the difference between the assigned value and the reference value is smaller than the experimental tolerance values of the atoms, which are 0.03 ppm for ^1^H, 0.4 ppm for ^13^C, and 0.4 ppm for ^15^N. Assignments outside this range are marked as wrong. Numbers in the parentheses are the percentages of correct, wrong, and unassigned chemical shift assignments relative to the total number of chemical shift values in the reference lists.

**Table 6:** Chemical shift assignment results for the Sso7d protein.^1.^

	NSGA2	NSGA3	GA	FLYA	PINE
Correct	456 (67)	37 (9)	381 (53)	588 (82)	607 (84)
Wrong	265 (33)	684 (91)	340 (47)	42 (6)	67 (9)
Unassigned	0	0	0	91 (13)	47 (7)
Run time (h)	6.8	6.9	4.8	3.3	0.1

^1^ The total number of correct, wrong, and unassigned atoms are given. The numbers in parentheses show the corresponding percentages relative to the total number of chemical shift values in the reference lists. See footnotes of Table 2 for details.

## Data Availability

Software and data are available at DOI 10.5281/zenodo.4707721 and https://github.com/emelmdn/ChemicalShiftAssignment (accessed on 12 June 2021).

## Sample Availability

Not available.
